# Review and Extension of CO_2_-Based Methods to Determine Ventilation Rates with Application to School Classrooms

**DOI:** 10.3390/ijerph14020145

**Published:** 2017-02-04

**Authors:** Stuart Batterman

**Affiliations:** Environmental Health Sciences, School of Public Health, University of Michigan, Ann Arbor, MI 48109, USA; stuartb@umich.edu; Tel.: +1-734-763-2417

**Keywords:** ventilation, air change rate, carbon dioxide (CO_2_), schools, classrooms, indoor air quality

## Abstract

The ventilation rate (VR) is a key parameter affecting indoor environmental quality (IEQ) and the energy consumption of buildings. This paper reviews the use of CO_2_ as a “natural” tracer gas for estimating VRs, focusing on applications in school classrooms. It provides details and guidance for the steady-state, build-up, decay and transient mass balance methods. An extension to the build-up method and an analysis of the post-exercise recovery period that can increase CO_2_ generation rates are presented. Measurements in four mechanically-ventilated school buildings demonstrate the methods and highlight issues affecting their applicability. VRs during the school day fell below recommended minimum levels, and VRs during evening and early morning were on the order of 0.1 h^−1^, reflecting shutdown of the ventilation systems. The transient mass balance method was the most flexible and advantageous method given the low air change rates and dynamic occupancy patterns observed in the classrooms. While the extension to the build-up method improved stability and consistency, the accuracy of this and the steady-state method may be limited. Decay-based methods did not reflect the VR during the school day due to heating, ventilation and air conditioning (HVAC) system shutdown. Since the number of occupants in classrooms changes over the day, the VR expressed on a per person basis (e.g., L·s^−1^·person^−1^) depends on the occupancy metric. If occupancy measurements can be obtained, then the transient mass balance method likely will provide the most consistent and accurate results among the CO_2_-based methods. Improved VR measurements can benefit many applications, including research examining the linkage between ventilation and health.

## 1. Introduction

The ventilation rate (VR) affects indoor environmental quality (IEQ) and the energy consumption of buildings. Ventilation with outdoor air is intended to remove moisture and pollutants emitted from indoor sources, and sufficiently high VRs are needed so as to not compromise IEQ and cause health, comfort, absenteeism and productivity problems [1,2,3,4,5,6,7,8,9,10]. Buildings are large contributors to energy consumption and greenhouse gas emissions, and heating, ventilation and air conditioning (HVAC) systems are the most demanding component among building energy services, accounting for an estimated 50% of building energy consumption and 20% of total energy consumption in the U.S. [11]. Excessively high VRs increase energy consumption and incur additional energy-related costs, however, the health benefits of higher VRs may greatly outweigh these costs [5]. In schools, for example, increasing the VR to be consistent with minimum recommended levels has been estimated to decrease absences related to illness and to yield a net economic benefit [6]. 

VRs should be consistent with minimum targets specified in building codes. Outdoor air rates specified for classrooms in ANSI/ASHRAE Standard 62.1 are the sum of personal (5 L·s^−1^·person^−1^) and area (0.6 L·s^−1^·m^−2^) minimum values [12]. Using the default occupant densities of 25 and 35 persons per 100 m^2^ for ages 5 to 8 and 9 and older, respectively, noted in the standard, the minimum VRs are 7.4 and 6.7 L·s^−1^·person^−1^ for the two age groups. With a ceiling height of 3 m, this is equivalent to air change rates of 2.22 and 2.01 h^−1^. For residential dwelling units, the same standard prescribes 2.5 L·s^−1^·person^−1^ and 0.3 L·s^−1^·m^−2^. For a 200 m^2^ area, 600 m^3^ volume, 3-bedroom dwelling with 4 occupants, the VR is equivalent to 20 L·s^−1^·person^−1^ and an air change rate of 0.48 h^−1^, similar to a minimum of 0.5 h^−1^ recommended for homes [5]. 

Ventilation metrics, including the VR (m^3^·h^−1^), VR per person (L·s^−1^·person^−1^), the outdoor air change rate (A, h^−1^) and others [13], can be determined using air flow measurements [14], pulse or constant injections of tracer gases [15,16,17,18], occupant-generated carbon dioxide (CO_2_) as a “natural tracer” gas [19,20,21,22,23,24,25,26], and the comparison of indoor and outdoor concentrations, concentration trends, and sometimes temperatures [27,28]. Air flow measurements from fan pressurization tests [29] provide a related measurement, the “tightness” of the building envelope. A recent review describing the use and history of air change rate measurements highlights the use of the constant tracer injection method with perfluorocarbon tracers like SF_6_ [13]; this method also allows multizone analyses [30]. VRs are measured for many purposes, e.g., to inform complaint-driven building investigations, to verify building system performance (e.g., commissioning), and as part of epidemiologic studies. However, obtaining accurate VR measurements can be challenging [19], and relatively few IEQ studies have adequately measured VRs or otherwise appropriately characterized the ventilation performance of buildings under study [13]. Additional information is needed to improve the understanding of the linkage between ventilation and health [31]. 

Occupant-generated CO_2_ has been widely used as a tracer gas for estimating the VR. CO_2_-based methods are convenient since CO_2_ is inert, emission sources (people) are present in all buildings and usually well dispersed throughout occupied spaces, and inexpensive and reasonably accurate measurement and logging instrumentation is available. CO_2_ is especially suitable for high occupancy spaces like schools since indoor levels can far exceed outdoor concentrations [32]. In addition, occupant-generated CO_2_ does not raise issues associated with the injection of a tracer gas, especially a potent greenhouse gas like SF_6_, that can concern school officials and require parental permission. CO_2_-based methods have been classified by the occupancy phase or the concentration trend into build-up (or “step-up” or “charge-up”), steady-state (or equilibrium), and decay (or “step-down”) phases [15,25,32]. Other CO_2_-based methods include pulse injections, techniques that combine multiple occupancy phases, and transient mass balance methods that can model multiple occupancy phases as well as arbitrary occupancy patterns. Guidance and standards for several of these methods is available [24,26,33]. These methods have different assumptions, strengths and limitations [24,25] and, as shown later, can produce different results. 

While convenient, CO_2_ is not an ideal tracer gas [24]. CO_2_ sources are not unique, e.g., replacement air contains CO_2_ whether it is outside air where the global level currently averages 400 ppm, or if replacement air arises from other portions of the building. In addition, outdoor CO_2_ levels fluctuate daily and diurnally, e.g., levels tend to be lowest in the early afternoon and highest in the early morning with average diurnal changes from 50 to 100 ppm in large urban areas such as Baltimore, and levels vary by location, largely due to traffic-related emissions that can produce yet larger increments over the global level [34]. Also, CO_2_ generation rates in buildings vary over time and the time window considered for VR measurements, depending on the number of occupants and the level of metabolic activity. Generation rates are rarely measured, but instead estimated based on relationships established between occupant weight, height (or age and gender) and metabolic activity. Finally, sensor performance can be a concern. While the non-dispersive infrared (NDIR) sensors commonly used to measure CO_2_ are considered stable, durable and robust against interferences from other air components and pollutants, these sensors can be affected by temperature, atmospheric pressure and length of use [35]. 

This paper reviews and critiques CO_2_-based methods for estimating the VR with specific application to school classrooms. We present techniques presented in the literature, propose enhancements to the build-up method, extend the transient mass balance method, provide school grade- and age-specific emission rates needed for these methods, and discuss the effect of post-exercise recovery periods on CO_2_ generation. The methods are demonstrated for classrooms in four schools. Because the literature contains very few if any reports that compare CO_2_-based VR methods to reliable or “reference” methods for relatively large buildings like schools under real-life conditions, a difficult task but a clear gap in the literature, this review focuses on a comparison of the CO_2_-based methods. The paper consolidates methods and experience in the literature, and provides guidance for applications in classrooms, a critical environment with known ventilation issues.

## 2. Materials and Methods 

### 2.1. Approaches to Estimating Ventilation Rates

Methods using CO_2_ as a tracer gas are based on a fully mixed mass balance model:

(1) $$V\;\frac{\mathrm{dC}}{\mathrm{dt}}=E+{Q\;C}_{R}-Q\;C$$

where V = room (or zone) volume (m^3^); C = CO_2_ concentration in the room (mg·m^−3^); C_R_ = CO_2_ concentration in outdoor air or replacement air (mg·m^−3^); Q = flow rate of outdoor or replacement air (m^3^·h^−1^), and E = CO_2_ emission rate of indoor sources (mg·h^−1^). Generally, E is calculated as n G_P_, where n = number of persons in the space, and G_P_ = CO_2_ generation rate per person (L·h^−1^), which is age- and activity level-specific (as described later). The air change rate, A (h^−1^), is Q/V. Concentrations throughout the zone are assumed to be equal; this should be confirmed using measurements at multiple locations [24]. This assumption can be violated by the uneven distribution of CO_2_ sources and by limited air distribution effectiveness. The CO_2_ methods discussed in this paper apply only to a single and fully mixed zone. Also, VRs derived using CO_2_-methods will include outdoor air delivered via both the ventilation system and infiltration. Finally, most applications of tracer gas methods assume that the VR is constant over a specific time window, i.e., the period over which the CO_2_ concentration trend or peak level is measured and analyzed. 

The outdoor air flow rate per person, V_0_ (L·s^−1^·person^−1^), is obtained from air flow rate Q (m^3^·h^−1^) or A (h^−1^):

V_0_ = 0.2778 A V/n = 0.2778 Q/n (2)

where the constant accounts for the volume and time conversions. If the number of occupants varies over the time when the VR is determined, then the choice of n is critical. In schools, students and staff often leave for lunch, recess and for other reasons, thus the average occupancy over the school day can be much lower than the maximum occupancy. In consequence, V_0_ determined using the average occupancy is higher, and often considerably (by about 50%) than that based on the maximum occupancy. To assess the adequacy of ventilation by reference to VR guidelines expressed as L·s^−1^·person^−1^, the use of the maximum occupancy appears most consistent. To understand contaminant exposures, the use of the average occupancy is preferable. This difference does not appear to have received attention in the literature, possibly because prior studies have not had continuous occupancy information, however, this issue appears important based on our recent experience in school classrooms [36].

VRs in both naturally- and mechanically-ventilated buildings can be affected by time-varying factors including internal heating and cooling loads, outdoor temperature and the indoor-outdoor temperature difference, and wind speed and direction [37]. In buildings using variable air volume (VAV) systems, air flow and the VR will depend on thermal load. If the VR varies in the study time window used to estimate the VR, then the assumption of a constant VR required by most of the CO_2_-based methods will be violated, although the VR estimate may be useful if the variation is small. 

### 2.2. Steady-State Methods

Steady-state or equilibrium methods apply after CO_2_ levels have reached a steady-state concentration. The method is described by guidance and standards [16,24,33]. The steady-state air change rate, A_S_ (h^−1^), is calculated as:

A_S_ = 6 × 10^4^ n G_P_/{V (C_S_ – C_R_)} (3)

where n = number of persons in the space; G_P_ = average CO_2_ generation rate per person (L·min^−1^·person^−1^); V = volume of the room or space (m^3^); C_S_ = steady-state indoor CO_2_ concentration (ppm); and C_R_ = CO_2_ concentration in replacement or outdoor air (ppm) (Given the widespread practice, the remainder of this paper uses concentration units of ppm). Steady-state methods assume that the CO_2_ generation rate (i.e., the number and physical activity of occupants) over the study time window is constant for a sufficiently long period to reach the indoor equilibrium concentration C_S_. (If C_R_ changes over the time window, then the difference between C_S_ and C_R_ should approach a steady-state level.) ASTM [24] suggests that the measured C_S_ should reflect at least 95% of the equilibrium value (i.e., as attained after three complete air changes), and provides comprehensive guidance for this method including methods to estimate uncertainty. As noted, steady-state methods assume the VR is constant over the study time window, and most assume that the outdoor CO_2_ concentration is constant.

In practice, the average age and average activity level of occupants are used to estimate G_p_, the replacement air concentration C_R_ is preferentially measured [24] or assumed to be 400 ppm [38], and C_S_ is determined as the maximum 5- to 20-min average concentration over the study time window [1]. Some spaces may not reach steady-state conditions over the workday or study period. For this reason, the steady-state method is not recommended in schools if classes last 45 min or less and the air change is below 4 h^−1^ [21,25]. Many schools have much lower VRs, thus, the number of occupants must be constant for at least several hours to approach steady-state levels. If occupancy varies around the time of the peak concentration, then the method’s assumptions are not met. In classrooms, averaging the number of occupants n and the generation rate G_P_ just prior to the observed CO_2_ peak may avoid anomalies if the occupancy fluctuates widely. 

Steady-state methods may be used to estimate the VR per person using Equation (2) or as:

V_0,S_ = (1.67 × 10^4^) G_P_/(C_S_ – C_R_) (4)

where V_0,S_ = outdoor air flow rate per person (L·min^−1^·person^−1^), and the constant converts the generation rate from hours to seconds and concentrations C_S_ and C_R_ from ppm to a mixing ratio. For example, using a generation rate for a moderately active adult (1.7 MET; G_P_ = 0.46 L·min^−1^·person^−1^ (Section 2.5), C_S_ = 1479 ppm, and C_R_ = 400 ppm, Equation (4) gives V_0,S_ = 7.1 L·s^−1^·person^−1^, the minimum VR recommendation for many indoor spaces, including classrooms [12]. 

### 2.3. Decay Methods

Decay or step-down methods can be used when a space is vacated after occupancy, or if there is a stepwise decrease in occupancy [16,24]. The decay air change rate, A_D_ (h^−1^), for a single (and well-mixed) zone can be estimated using two CO_2_ measurements:

A_D_ = 1/Δt ln{(C_1_ − C_R_)/(C_0_ − C_R_)} (5)

where Δt = period between measurements (h); C_0_ and C_1_ = measured CO_2_ concentrations over the decay period (ppm); and C_R_ = CO_2_ concentration (ppm) in replacement air or the steady-state concentration at the lower occupancy in the case of stepwise decrease in occupancy. The stability of this 2-point estimate can be checked using an upper bound estimate of A_D_, obtained by selecting C_1_ and C_0_ as the maximum and minimum concentrations, respectively, occurring near the nominal times specified, e.g., within ±1 h.

Alternatively, a sequence of CO_2_ concentrations over a portion of the decay period, C_t_, may be used to fit a solution to Equation (1) using regression or other means:

C_t_ = (C_S_ − C_R_) exp(−A_D_ t) + C_R_ (6)

where t = time of the measurement (h). Equation (6) can be linearized:

ln(C_t_ − C_R_) = −A_D_ t + ln(C_S_ − C_R_) (7)

where C_S_ = the steady-state CO_2_ concentration. The estimated decay air change rate A_D_ is the slope of the regression of ln(C_1_ − C_R_) against time t. The regression intercept is ln(C_S_ − C_R_), thus C_S_ is equal to exp(intercept) + C_R_. 

Decay methods have been used to estimate VRs in schools [21,32,39,40,41]. The decay method is simple, and the regression approach does not require knowledge of C_R_, C_S_, G_P_, *n* or even V. However, there are important caveats. First, the appropriate time window for analysis can require careful selection [39,42]. The concentration change over the period must be large relative to the variation in C_R_ and CO_2_ measurement error; typically, changes of 100 ppm or more may be sufficient, but at least several hundred ppm are desirable given the performance of typical instrumentation. (Many CO_2_ instruments report accuracies of ±50 ppm plus 1% to 2% of readings.). Second, while CO_2_ decay curves often display near ideal behavior in many spaces, in rooms that utilize natural ventilation, opening windows during class breaks can overestimate VRs during classes [32]. Conversely, if windows are opened during occupancy but closed afterwards, VRs will be underestimated. Opened windows also can lead to significant variation in concentrations in the space, thereby violating the well-mixed assumption. Third, in mechanically-ventilated school buildings, while windows are rarely opened (and sometimes not openable), HVAC systems typically are shut down at the end of the school day. Because of the shut-down, decay air change rates will not apply to the occupied portion of the day. This applies to most U.S. schools where shut-down may occur immediately following the last class, e.g., as early as 14:20 (2:20 p.m.). (24-h time notation is used throughout this paper.) Fourth, VAV systems will provide less ventilation air if the thermal load diminishes after the space becomes unoccupied, which would have the effect of lowering the VR during the decay period even if the HVAC system is not shutdown. This especially applies to densely occupied spaces, including some classrooms. Fifth, in both naturally- and mechanically-ventilated buildings, VRs and/or infiltration rates will depend on the indoor-outdoor temperature difference, wind speed, heating and cooling load, and other factors that may change over time. For these reasons, the decay-based VR may not reflect conditions during the (occupied) school day, although it may provide information regarding the infiltration rate. Finally, while both the two-point and multipoint methods provide identical results under idealized circumstances, the former is more sensitive to measurement error and thus may be less accurate. 

### 2.4. Build-Up Methods

Build-up methods use the increase in CO_2_ concentrations following occupancy to determine VRs with the assumptions that the CO_2_ generation rate and VR are constant over the study time window, and the zone is fully mixed (The assumption of a constant CO_2_ generation rate in classrooms is discussed in Section 2.5). Build-up methods can estimate the VR just after a building is occupied, or after a step-wise increase in occupancy. The method may be preferable to both the steady-state and decay methods since the derived VR applies to the occupied period, and since steady-state conditions are not required. 

There are many approaches to solving the single zone mass balance for the build-up period. One approach calculates the build-up air change rate A_B_ (h^−1^) using two sequential CO_2_ measurements:

A_B_ = 1/Δt ln{(C_S_ − C_0_)/(C_S_ − C_1_)} (8)

where Δt = period between C_0_ and C_1_ measurements (h), C_S_ = steady-state concentration (ppm), and C_0_ and C_1_ = CO_2_ concentrations measured at start and end of the observation time window, respectively (ppm). C_S_ may be derived in several ways. A 3-point method uses a third concentration, C_M_, measured at the midpoint in time between the C_0_ and C_1_ measurements [25]. If the build-up curve follows the expected approach for the method’s assumptions (i.e., smooth and initially rapid increase that gradually plateaus), and if the method’s assumptions are valid, then Ĉ_S_ may be estimated as:

Ĉ_S_ = (C_M_^2^ – C_0_ C_1_)/(2 C_M_ – C_0_ − C_1_) (9)

Appropriate times for measuring C_0_, C_1_ and C_M_ in classrooms and other applications will depend on occupancy patterns and building system operation. Because build-up curves may not follow the expected approach due to time-varying occupancy or other reasons, Equation (9) can be sensitive to the time period selected (e.g., the estimated C_S_ can be negative and meaningless if C_M_ is less than the average of C_0_ and C_1_). 

A second solution technique for the build-up method is proposed that circumvents some of the issues associated with the 3-point method. This uses an estimate of C_S_ obtained by simultaneously solving Equations (3) and (8) so that A_S_ = A_B_. Using the average number of occupant n (persons), age-adjusted CO_2_ generation rate G_P_ (L·min^−1^·person^−1^), zone volume V (m^3^), replacement air concentration C_R_ (ppm), and an initial air change rate estimate denoted as Â_B_ (h^−1^), an initial estimate of the steady-state concentration Ĉ_S_ follows from Equation (3):

Ĉ_S_ = 6 × 10^4^ n G_P_/(V Â_B_) + C_R_ (10)

By substituting Equation (10) into Equation (8) and simplifying, Â_B_ can be solved for as the root of the following equation:

0 = exp(Â_B_ Δt) − {(6 × 10^4^ n G_P_/(V Â_B_) + C_R_ − C_0_)}/{6 × 10^4^ n G_P_/(V Â_B_) + C_R_ − C_1_} (11)

While no analytical solution exists, Equation (11) can be solved by numerical root-finding methods. Because Equation (11) has local minima and inflection points, a robust algorithm and an appropriate starting solution should be used. Since C_S_ must exceed C_1_, Â_B_ is bounded and an upper bound estimate (that can be reduced slightly to use as a starting estimate) is:

Â_B,0_ < 6 × 10^4^ n G_P_/{V C_1_ (1 − C_R_/C_1_)} (12)

Equation (11) was solved using a modified Newton-Raphson method (described in Supplementary Materials). This implicit solution to the build-up method will yield results identical to the 3-point or other (exact) build-up methods under ideal circumstances. However, it may be more stable and less sensitive to the time window selected. It somewhat resembles a steady-state method encompassing a “correction factor” to account for measurements taken prior to reaching steady-state conditions [26]. 

A third solution method can be adopted from the ASTM E471 Standard, which includes a build-up method for a continuous injection of a tracer gas [16]. This is adopted for CO_2_ by subtracting C_R_ from the series of CO_2_ observations measured over the study period, C_t_ at times t = 1 … T, expressing concentrations in ppm, and calculating the air change rate (rather than the air flow):

A_B_ = 6 × 10^4^ n G_P_ {Σ_t_ (C_t_ − C_R_)^−1^}/(V T) − Δt^−1^ ln{(C_1_ – C_R_)/(C_0_ – C_R_)} (13)

where T = number of measurements in the summation, i.e., the expression T^−1^ Σ_t_ (C_t_ − C_R_)^−1^ is the average of the inverse of the CO_2_ measurements after subtracting C_R_. The first term in Equation (13) is equivalent to the steady-state solution shown as Equation (3), but with the use of multiple measurements; the second term provides a “correction” given that concentrations C_0_ and C_1_, taken at the beginning and ending of the study time window, respectively, are not at steady-state. This solution to the non-steady-state problem can be sensitive to the time window selected, e.g., using the early part of the build-up curve can inflate the summation term due to relatively large values of inverse C_t_, which leads to an overestimate of A_B_.

A fourth solution technique for the build-up VR also uses the series of concentration measurements C_t_ over the build-up period with a solution to the fully mixed model

C_t_ = (C_S_ − C_R_) {1 − exp(−A_B_ t)} + C_R_ (14)

which may be linearized:

ln(C_S_ − C_t_) = −A_B_ t + ln(C_S_ − C_R_) (15)

allowing A_B_ to be obtained as the (negative) slope of ln(C_S_ − C_t_) versus time t. The regression intercept is equal to ln(C_S_ − C_R_), thus, C_R_ is estimated as C_S_ – exp(intercept). This approach requires an estimate of C_S_, along with the sequence of CO_2_ measurements. A fifth and related solution method might use a non-linear solver to simultaneously estimate A_B_ and C_S_ in Equation (15), e.g., by minimizing the squared residuals. 

Only a few applications of the build-up method in schools were identified [43,44]; these appear to have used the 3-point method (Equations (8) and (9)) to estimate C_S_. The build-up method may be used with relatively short occupied classroom periods and with low air change rates [32]. 

Build-up methods can be sensitive to the time window selected, as noted. In U.S. schools, the school day in primary and secondary schools generally starts about 07:30 to 08:00 and lasts till about 14:30 or 15:00, however, there can be considerable variation and many classrooms have substantial drops in occupancy during midday (e.g., due to lunch), in the afternoon, and at other times. In addition to sensitivity to the time window, the build-up air change rate may be more affected by incomplete mixing than the decay method [32]. The method assumes a constant CO_2_ generation rate, which requires that the number of occupants in the space and level of physical activity are unchanged over the time window, assumptions that are examined in Section 2.7. Finally, while the various solution techniques for the build-up method can provide identical results under idealized circumstances, the three point method requires that concentrations closely follow the expected trend; this method as well as the implicit method can be sensitive to measurement error; the implicit method also depends on the accuracy of the occupancy and emission estimates; and the ASTM method requires near-steady-state conditions to minimize errors. In contrast, the multipoint solution method using Equations (14) and (15) is potentially more robust. These issues are explored in Section 3.

### 2.5. Transient Mass Balance Methods

Transient mass balance methods to determine the VR use single or multiple occupancy phases, time-resolved occupancy and CO_2_ observations, and a numerical solution to the mass balance model, Equation (1). An approximate but useful solution is:

C_t + 1_ = 6 × 10^4^ n_t_ G_P_/Q {1 − exp(−Q/V Δt)} + (C_t_ − C_R_) exp(−Q/V Δt) + C_R_ (16)

where C_t_ = observed CO_2_ concentration at time t (ppm); n_t_ = sequence of occupancy rate observations at time t (persons); G_P_ = average CO_2_ emission rate (L·min^−1^·person^−1^); Q = replacement air flow rate (m^3^·h^−1^), V = volume of the space (m^3^); C_R_ = replacement air CO_2_ concentration (ppm); and Δt = time interval for CO_2_ and occupancy observations (h). The estimated air change rate, A_TMB_ (h^−1^), is Q/V. The CO_2_ build-up from emission sources present in the space during the period Δt is expressed as the first exponential term, and the CO_2_ decay as the second term. If flow rate Q is constant, then the expression exp(−A/V Δt) in both terms is constant. Equation (16) is exact for step-wise changes in occupancy at Δt intervals (as obtained by occupant survey data). The key unknown, Q, is determined as the value that meets an error criterion, e.g., a numerical solver may be used to fit Q by minimizing the sum of squares between predicted and observed concentrations. We also recommend fitting the CO_2_ concentration at the beginning of the study time window using constraints, e.g., minimum of 375 ppm. Estimating this concentration, rather than selecting the CO_2_ measurement, will increase model fit and may avoid issues associated with an anomalous measurement, minor sensor errors, and in cases, CO_2_ levels above C_R_ that remain elevated from the previous day. The term exp(−Q/V Δt), which does not change with time, may be precomputed to increase the optimization speed. 

The feasibility of using transient mass balance methods with CO_2_ to estimate VRs was shown for a university library in England in 1980 [45]; more recently, the method was used to determine outdoor air flow rates per person (V_0_) in 16 classrooms in nine schools (seven naturally ventilated) in England [46]. Applications of the method using tracer gases other than CO_2_ may be more common, e.g., the method was used with SF_6_ to determine VRs in 62 naturally ventilated classrooms in 27 schools in Greece; results showed strong correlation between VRs and CO_2_ concentrations [47]. CO_2_ simulations have been used to evaluate VRs in a few mechanically- and naturally-ventilated school rooms in the U.S. [22]. Smith [23] presents additional analyses, including extensions to multizone applications. Methods to estimate uncertainty using transient methods have been discussed elsewhere [23,45]. 

The transient mass balance method is very flexible. It does not require steady-state conditions, and it can be used with arbitrary (but known) occupancy patterns. It can estimate the VR for different portions of the day and different occupancy phases, e.g., morning, afternoon and evening periods, and include occupied and unoccupied periods, either separately or combined. (However, periods that might have different VRs should be analyzed separately.). The method has less sensitivity to the time window selected, any time interval Δt can be used, and the replacement air concentration C_R_ can vary in time (if known). In addition, other unknown or uncertain parameters can be estimated, e.g., the replacement air concentration C_R_ and the metabolic activity level used to determine G_P_ (as well as the initial CO_2_ concentration mentioned). Such applications, however, should constrain estimated variables to ensure plausible values, aid convergence, and limit the sensitivity of results. While application-specific parameters should be used, for example, C_R_ if estimated might be constrained to the range of 350 to 500 ppm, reflecting measurement uncertainty of ±50 ppm and an urban increment over average global levels of 50 ppm. Potentially the method can be used to account for the post-exercise recovery period that may temporally increase G_P_ (see Section 2.5). Finally, fitting criteria can be calculated as a quality check, e.g., a minimum fraction of variance explained (R^2^) might be required, thus ensuring acceptable agreement between predicted and observed CO_2_ concentrations.

### 2.6. CO_2_ Generation Rates 

The CO_2_-based VR methods, other than the decay method and some forms of the build-up method, require estimates of the CO_2_ generation rate G_P_, and all VR methods assume that G_P_ is constant (or that its variation is known) over the time window used for analysis. This section uses a method documented elsewhere [24] to calculate school grade-level specific rates suitable for classroom applications. In addition, we include updated physical activity estimates and consider the post-exercise recovery period that can temporarily increase G_P_. 

Estimates of G_P_ can be derived using relationships established between occupant weight, height (or age and gender) and metabolic activity. The Dubois equation estimates an individual’s skin area:

S = 0.20247 H^0.725^ W^0.425^ (17)

where S = skin area (m^2^); H = height (m); and W = weight (kg) of an individual. The CO_2_ generation rate per person, G_P_ (L·min^−1^·person^−1^), is the O_2_ consumption rate multiplied by the respiratory coefficient (0.83):

G_P_ = (60) (0.83) (0.00276) S MET/{(0.23) (0.83) + (0.77)} (18)

where MET = metabolic equivalent, a measure of an individual’s energy expenditure of physical activities. 

Height and weight data of children for Equation (17) come from U.S. representative growth charts that list sex-specific data at the monthly level (Table 1) [48]. We took the 12-month average by grade level (assuming 1st grade children are between 6 and 7 years of age, 2nd grade between 7 and 8 years of age, and so on). For teachers, height and weight data for adults aged 20 to 70 years use representative U.S. statistics derived from NHANES 1999–2006 (Table 1) [49]. The relative variation in these data were calculated as the interquartile range (75th − 50th percentile) divided by the median (in percent). For height, this relative variation was 6% for both boys and girls, and 5% for adults. For weight, the relative variation was 22% and 30% for boys and girls, respectively, and 26% and 32% for men and women, respectively. 

Physical activity levels depend on task and intensity, and standard MET-based definitions have long been used to classify physical activity levels into sedentary (<1 to 1.5 MET), light (1.5 to <3 MET), moderate (3 to <6 MET) and vigorous (>6 MET) classifications. For youth (8–18 years of age), recent analyses suggest that resting energy expenditures exceed adult METS by about 33%, thus increasing the ranges stated; consequently, sedentary behaviors of children may range of up to 2.0 MET [50]. The recent compendium of physical activity for children most commonly lists 1.4 MET for children sitting quietly, studying, taking notes and writing, and having class discussion [51]. This value exceeds the “typical” MET levels stated in the guidance for using CO_2_ for ventilation evaluations, i.e., 1.0 to 1.2 MET for reading, writing and typing while seated [24]. For adults, the most recent compendium lists 1.5 MET for sitting tasks with light effort, e.g., office work, reading, computer work and talking; standing tasks with light effort, e.g., directing traffic, filing, talking and teaching physical education, range from 2.0 to 3.0 MET [52]. Again, these exceed values in the guidance noted earlier (1.0 to 1.2 MET for sitting activities noted earlier, and 1.4 and 2.0 MET for filing while standing and walking, respectively). Teachers and other adults in classrooms are expected to engage in a combination of sitting, standing and walking activities, thus a blend of sedentary and light activity levels is appropriate, e.g., 1.7 MET may be appropriate for a teacher who occasionally stands and walks about the classroom. 

Using a physical activity value of 1.4 MET and averaging across boys and girls, the CO_2_ generation rate G_P_ in classrooms ranges from 0.147 L·min^−1^·person^−1^ for pre-kindergarten children to 0.343 L·min^−1^·person^−1^ for 12th graders (Table 1). Several studies have used a single generation rate for 5th grade children, equivalent to 0.258 L·min^−1^·person^−1^ [1,6,8]; the strong dependence on age is important to recognize. For women, using a physical activity value of 1.7 MET and averaging across ages, G_P_ is 0.442 L·min^−1^·person^−1^. G_P_ estimates for adults have little sensitivity to age (<3% variation), unlike for children. The assumption of female teachers, used since most teachers (88%) in our school surveys have been women, is not significant since the total CO_2_ generation rate in classrooms is usually dominated by students. The total classroom emission rate can be calculated as the sum of emissions from children (grade level-specific G_P_ multiplied by the number of students present) and adults (adult G_P_ multiplied by the number of adults present). 

The variability of the population’s height and weight can affect G_P_, as demonstrated using a limited sensitivity analysis. Using the relative variability described earlier and examining 6th grade children, a 6% increase in height over the nominal case (Table 1) would increase G_P_ by 4% (average for boys and girls); 22% and 30% increases in weights of boys and girls, respectively, would increase G_P_ by 10%, and increasing both height and weight by these amounts would increase G_P_ by 15%. In most classrooms with a mix of children and imperfect correlation between height and weight, uncertainties due to height and weight variation are expected to be smaller. Uncertainties related to physical activity and metabolism levels, discussed below, are likely much more significant. 

In most applications, the physical activity level of building occupants is unknown, and the actual value might considerably exceed the assumed level. Also, if the activity level changes, the assumption of a single and constant activity level might not adequately represent the CO_2_ generation rate G_P_. In addition, energy expenditures of occupants depend on activity levels prior to entering the space. In schools, for example, students may enter a classroom following more vigorous activity, e.g., walking/running to class, playing at recess, etc. Factors that affect the post-exercise recovery time needed to reach resting (or sedentary) metabolism levels include the duration and intensity of the preceding exercise and the individual’s condition and age, e.g., recovery periods for children are about half that of adults [53]. Information relevant to previous activity levels and the corresponding G_P_ pertaining to students entering a classroom in the recovery period is not directly available, but can be inferred from several sources. For example, guidance for measuring the resting metabolic rate in adults in clinical settings suggests a minimum rest period of 10 to 20 min, and an abstention period of 2 h following moderate aerobic or anaerobic exercise [54]. The duration of excess post-exercise oxygen consumption (EPOC) for short and low intensity exercise across a number of studies examining adults listed in a comprehensive review of EPOC studies ranged between 6 and 30 min [55]. Comparable EPOC studies for healthy children engaged in low intensity exercise relevant for schools were not identified.

To evaluate effects of prior physical activity, we analyzed several scenarios that considered children exercising just prior to entering a classroom. For exercise, we assumed walking with light to hard effort, equivalent to 2.9 to 4.6 MET [51], and a nominal value of 3.5 MET. For recovery periods, we assumed a recovery time t_REC_ (h) from 0.1 to 0.5 h, and a nominal period of 0.25 h (only adult values were available). The literature often represents recovery trajectories as a first-order (exponential decay) trend, but recovery durations have been reported using different methods. We assumed that the reported recovery time reached the resting metabolism rate within 5% of the pre-exercise value, and thus the recovery time rate constant K_REC_ (h^−1^) = ln(0.05)/t_REC_ (Larger percentages, 10% and 15%, were are also tested). With these assumptions, an individual’s instantaneous activity level after entering a classroom, MET_t_, will exponentially decline to the sedentary level, MET_SED_:

MET_t_ = (MET_EXER_ – MET_SED_) exp(−K_REC_ t) + MET_SED_ (19)

where MET_EXER_ = physical activity level during exercise (MET); and t = time elapsed since entering the classroom and the cessation of exercise (h). To gauge the importance of the recovery effect over the CO_2_ monitoring time window, MET_t_ is averaged over the time period the student is in the classroom and compared to MET_SED_ (assumed to be 1.4 MET), and expressed as a relative bias (%). This represents the amount of additional activity resulting from the post-exercise recovery period, and also the extent to which CO_2_ emissions will be underestimated if the post-exercise recovery effect is ignored. 

### 2.7. Application

Four classrooms in different primary schools were selected to demonstrate the VR methods, several of which have not been applied previous in schools, and to show a range of ventilation and occupancy conditions. The selected schools are a subset of those in a larger (Environmental Quality and Learning in Schools or EQUALS) study. These schools were located in the U.S. Midwestern states of Michigan, Illinois, Ohio and Indiana; all are mechanically-ventilated, and all were constructed or fully renovated in the past 15 years. Schools were visited and monitored in winter during the heating season (November 2015 to February 2016) when window or door opening was at a minimum. Our technicians conducted walk-through surveys of each classroom, ventilation system, school building, and grounds. Classroom dimensions were obtained using a laser tape measure. HVAC information, including system type, configuration, design and minimum air flow rates, were obtained from HVAC drawings, schedules and photos, as well as manufacturer’s specifications; this information was used to calculate VRs for comparison with those determined using the CO_2_-based methods. The studied spaces are summarized in Table 2. 

CO_2_ concentrations in each classroom were measured at a central location away from windows and doors using NDIR sensors (C7632A, Honeywell Corp., Morristown, NJ, USA); similar instrumentation on the school grounds or rooftop measured outdoor CO_2_ concentrations. CO_2_ data were reduced to 15 min averages. CO_2_ sensors were calibrated quarterly using zero air and a certified standard. Drift per season was typically below 25 ppm. Teachers in the selected classrooms were asked to maintain an occupancy log by recording the number of adults and children present every 15 min. This information was used to estimate CO_2_ generation rates on a 15 min basis using metabolic activity levels of 1.4 and 1.7 MET for children and adults, respectively, and the generation rates in Table 1. The effect of post-exercise recovery was not modeled, although a sensitivity analysis is performed in Section 3.3. One day with full data in each school was selected for analysis. To calculate build-up, steady-state and decay air change rates, study days were separated into several periods (see below) in which the VR was assumed to be constant. The 06:00 to 08:00 and 15:00 to 18:00 periods were excluded since the ventilation systems were turned on and off during these periods, which would change the VR. In addition, during these periods, occupancy rates changed dramatically, and the collected occupancy data may have been incomplete. 

Steady-state VRs were calculated using Equation (3), the maximum 15-min CO_2_ concentration over the school day (08:00 to 15:00), the measured room volume, the average generation rate over the 2 h prior to the time of the maximum CO_2_ concentration (determined from the 15-min average data), and 400 ppm for the measured outdoor CO_2_ concentration, which was confirmed using the outdoor measurements. V_0,S_ was calculated using Equation (4), the average per person CO_2_ generation rate in the classroom, and the number of persons in the classroom in the 2 h period just prior to the peak CO_2_ concentration. We determined the sensitivity to the time period and outdoor air concentration. 

Decay air change rates were estimated using Equation (5), 15-min average CO_2_ measurements measured at 18:00 and 24:00 for the “evening” period, and concentrations at 24:00 and 06:00 for the “early morning” period. We searched for the maximum and minimum concentrations occurring within ±1 h of the nominal period, which were used as C_0_ and C_1_ in Equation (5). Comparable decay air change rates were found using regression to fit Equation (7) (results not shown). In addition, transient mass balance air change rates were estimated for the same periods using the sequence of 15 min average CO_2_ measurements. 

Build-up air change rates were determined using the 2-point method in Equation (8) with an implicit estimate of C_S_ determined using Equations (10) and (11), C_0_ and C_1_ measured as 15-min averages, a starting estimate given as 0.9999 × Â_B,0_ from Equation (12), and the Newton-Raphson search method, described earlier (also see Supplemental Information). Given the occupancy patterns noted, we selected a nominal period from 08:00 to 12:00, but allowed actual start and end times to vary by ±1 h (as described earlier). The average CO_2_ generation rate over the selected time window was used to estimate C_S_. The build-up method was also implemented using the 3-point and ASTM methods (Equations (8), (9) and (13), respectively), the same selected times, and the 15-min concentration at the midpoint time (or the average of two consecutive 15-min concentrations given an even number of observations between C_0_ and C_1_). 

Transient mass balance air change rates were determined using Equation (16) for three periods: the school day (8:00 to 15:00 p.m.), an evening period (18:00 to 24:00), and an early morning period 24:00 to 06:00). We used an interval of 15 min (Δt = 15 min) for observed and simulated CO_2_ concentrations, and the CO_2_ generation rate (using teacher-reported occupancy data). In addition to A_TMB_, we estimated the replacement air concentration (C_R_) with constraints (375 ppm < C_R_ < 450 ppm), the children’s metabolic equivalent level within constraints (1.2 < MET < 1.6), and the CO_2_ concentration at the beginning of the simulation period with a constraint (C > 375 ppm). A generalized reduced gradient solver using central derivatives estimated these unknown parameters by minimizing the sum of squares between observed and simulated CO_2_ concentrations. VRs were estimated separately for each period. Each problem used the same initial solution. We also tested the sensitivity of results to the start and stop times and other parameters. Lastly, V_0_ is reported using both the average and the maximum number of occupants, and the estimated VR.

Outdoor temperatures and wind speeds measured at the airport nearest each school are shown in Table 2. During the school day (08:00–15:00), average temperatures varied considerably (−11 to 13 °C), depending on the day studied. Several days had moderately high wind speeds (up to 9 m/s for the school day average).

## 3. Results and Discussion

### 3.1. Comparison of Classrooms

**Figure 1:**
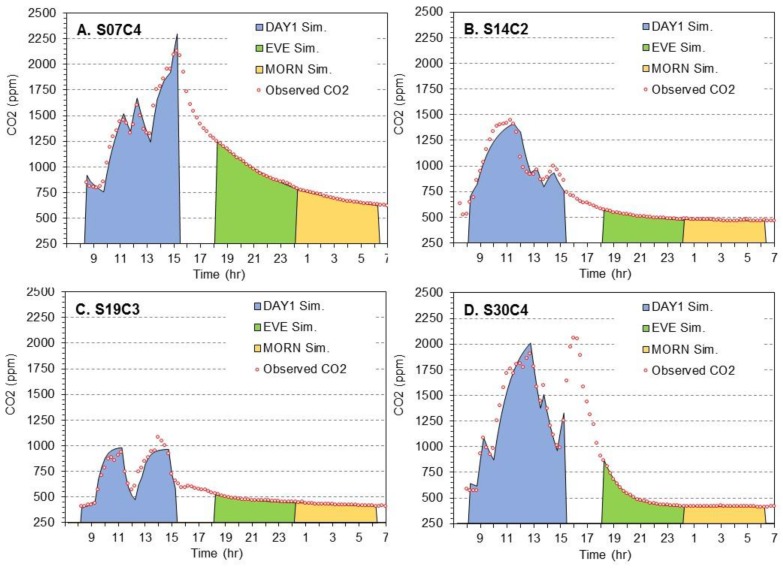
Observed and simulated CO_2_ concentration trends over 24-h periods in classrooms in two conventional school buildings (**A**,**B**) and two EnergyStar school buildings (**C**,**D**). Red circles show observed (15-min) levels; colored areas show predicted CO_2_ levels using simulated air change rate estimates fitted for the school day (blue), evening (green), and early morning (yellow) periods. Time axis shows hour of day (starting at 07:00).

Traces of observed and simulated CO_2_ levels over a 24-h period in the four classrooms are shown in Figure 1, and VRs determined using the four methods are listed in Table 3. Except where noted, predicted (using the transient mass balance method) and observed CO_2_ levels matched closely (R^2^ > 0.94). Results for the four classrooms are discussed in turn. 

Figure 1A shows trends for room S07C4. During the study period, the room contained as many as three adults and 41 children. Children were present continuously from 9:30 to 15:00 except for the lunch period (12:00 to 13:00), but the number of occupants fluctuated considerably, and the maximum occupancy occurred at the end of the school day when students from another class briefly joined the room (doubling the number of students). The “pointed” peak at the maximum CO_2_ concentration reveals that a steady-state level was not reached. During the school day (08:00 to 15:00), CO_2_ levels (15-min average) reached 2100 ppm, the transient mass balance air change rate was 0.51 h^−1^, the “steady-state” rate using the observed maximum CO_2_ level was 0.52 h^−1^, and build-up rates were 0.31, 0.43 and 0.52 h^−1^ for the 3-point, implicit and ASTM methods, respectively. All VRs calculated using CO_2_ fell well below those based on the HVAC schedule (shown in Table 2). Air change rates dropped to 0.14 h^−1^ in the evening (18:00 to 24:00) and then to 0.10 h^−1^ in the early morning (24:00 to 06:00). In the unoccupied period, CO_2_ trends followed the expected exponential decay, and the decay and transient mass balance methods gave comparable results. Based on the latter, V_0_ was 2.6 L·s^−1^·person^−1^ using the average occupancy (16.5 persons), but only 0.99 L·s^−1^·person^−1^ using the maximum occupancy (43 persons), an unusually large difference resulting from the large change in occupancy. CO_2_ levels remained above outdoor levels through the night and the next morning, a result of the previous day’s high CO_2_ levels and the low VR when the building was unoccupied and the HVAC system shutdown. 

Figure 1B shows results for S14C2. The teacher in this classroom often shut-off the UV (the only mechanical system in the room) due to noise and opened an outside door for ventilation. This classroom contained one adult and up to 20 children. The room was continuously occupied from 07:30 to 11:45, emptied for a 45 min lunch period, and then reoccupied for two smaller classes (with a gap in between) in the afternoon. During the school day, CO_2_ levels reached 1440 ppm, the transient mass balance and 3-point build-up VR estimates were both 0.77 h^−1^; the steady state and other build-up VR estimates were lower (Table 2). V_0_ was 5.5 L·s^−1^·person^−1^ using the average occupancy (12.6 persons) and 3.1 L·s^−1^·person^−1^ using the maximum occupancy (22 persons). During the (unoccupied) evening and early morning periods, VRs dropped 5- to 10-fold, and the transient mass balance and decay air change rates matched closely, although CO_2_ levels in the early morning had only small changes, which increased uncertainty and decreased fit (e.g., R^2^ = 0.82). Like Figure 1A, CO_2_ levels the following morning remained (slightly) elevated over outdoor levels, a result of a particularly low VR during this period.

Figure 1C displays trends in room S19C3, a classroom that contained one or two adults and 22 children for most of the morning. This room emptied at lunch, and then was re-occupied with about the same number of students in the afternoon. This pattern produced two “well-formed” CO_2_ build-up curves in the morning and in the afternoon that reached 1080 ppm. During the occupied period, the transient mass balance air change rate was 2.4 h^−1^, close to that estimated using the HVAC schedule (Table 2). The estimated V_0_ was 5.5 L·s^−1^·person^−1^ for the average occupancy (15.1 persons) and 9.5 L·s^−1^·person^−1^ for maximum occupancy (26 persons), the highest V_0_ among the four classrooms. The steady-state and 3-point build-up methods gave lower VRs; the ASTM build-up method gave a very high and spurious result, a result of initially low then rapidly rising CO_2_ levels. During the unoccupied evening and early morning periods, VRs dropped over 10-fold, e.g., transient mass balance and decay air change rates in the evening period were 0.27 and 0.17 h^−1^, respectively. The divergence between these VRs, larger than usual, reflects the low CO_2_ levels during the unoccupied periods that increased uncertainty (R^2^ = 0.88). 

Lastly, Figure 1D depicts 30C4. The room had one teacher and 27 students, but occupancy varied, i.e., most students left in the early afternoon, but about half were reported to have returned for their final class around 15:00. A second CO_2_ peak occurred in the late afternoon around 16:00 (this fell beyond the 15:00 cut-off used in the transient mass balance simulation). CO_2_ levels reached 1900 ppm in the morning and 2060 ppm in the afternoon. Like Figure 1A, the “pointed” peaks at the maximum CO_2_ concentration reveal that steady-state levels were not reached. For the occupied period, the transient mass balance air change rate was 0.71 h^−1^, the “steady-state” rate was 0.80 h^−1^, and again, the 3-point build-up rate was low and the ASTM build-up rate was high. The transient mass balance simulations capturing both peaks (running from 08:00 to 17:00, not shown) obtained a slightly air change rate (0.60 h^−1^), but the morning CO_2_ peak was over-predicted and the afternoon peak was under-predicted (both by about 300 ppm), suggesting that additional (and unreported) students had returned, that activity levels increased, or that the VR decreased. In this room, V_0_ was 3.3 L·s^−1^·person^−1^ using the average occupancy (16.6 persons) and 2.0 L·s^−1^·person^−1^ using the maximum occupancy. The evening trend in this classroom is unusual because the HVAC system was operated until midnight, thus the evening VR was relatively high (0.72 and 0.57 h^−1^ for the transient mass balance and decay methods, respectively). The air change rate dropped to very low levels (0.01 to 0.05 h^−1^) in the early morning when the HVAC system was shutdown.

### 3.2. Effect of Post-Exercise Recovery

**Figure 2:**
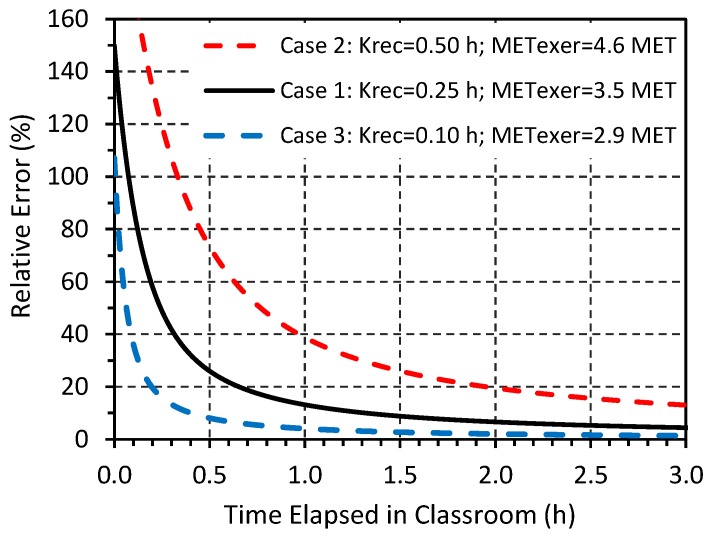
Effect of post-exercise recovery on CO_2_ generation and metabolic activity rates, expressed as relative bias for periods of 0 to 3 h, compared to classroom activity of 1.4 MET. Assumes children undergoing light to moderate exercise (2.9 to 4.6 MET) prior to entering the classroom. Three cases shown: Case 1 uses nominal parameters; Case 2 uses “maximal effect” parameters; Case 3 uses “minimal effect” parameters.

The predicted bias due to the post-exercise recovery period for a student entering a classroom is shown in Figure 2. (Further information, including trends of activity levels in MET, are shown in the Supplemental Information.) For the nominal case, prior exercise increased the total amount of CO_2_ generated over a 1, 2 and 3 h period by 13%, 7% and 4%, respectively, compared to CO_2_ generated by the classroom activities alone (at 1.4 MET). For the “maximal effect” case (T_R_ = 0.5 h; MET_EXER_ = 4.6), the increase was 39%, 20% and 13%, respectively; for the “minimal effect” case (T_R_ = 0.1 h; MET_EXER_ = 2.9), the increase was only 4%, 2% and 1%. Relaxing the assumption that reported recovery times reached the resting metabolic rate reported in the literature to within 5%, specifically increasing this percentage to 10% and 15%, only modestly increased the estimated bias. 

While the analysis is approximate and uncertain given the data gaps (particularly those for children) and the simplified assumptions, results suggest that estimates of physical activity and thus G_P_ applying to a student entering in a classroom will be underestimated if the preceding exercise is of high intensity (>4 METS) and the measurement time window is short (<1 or 2 h) and immediately follows the students’ entry into the classroom. To significantly affect the total CO_2_ generation rate, this must apply to most students entering the classroom. In such cases, the recovery effect will be significant and G_P_ will be significantly underestimated, leading to an overestimate of the VR. The build-up method may be especially prone to the post-exercise recovery effect since it emphasizes the early portion of the CO_2_ trend; results for the transient mass balance and steady-state methods may also be affected, although smaller effects are expected since these methods tend to emphasize the higher CO_2_ concentrations that occur several hours after students enter the classroom. Figure 2 also suggests that the time window selected for analysis, if possible, should exclude the first hour after students enter the space. In many situations, however, the bias may be modest (<10%) even with considerable traffic into a classroom, a result of children’s quick recovery time (possibly underestimated since the analysis used the adult values), and since VR determinations typically use multihour-long time windows. 

Examination of CO_2_ trends in the sample classrooms did not show evidence of post-exercise recovery effect, although effects could be obscured by other factors. In particular, physical activity levels might not be elevated prior to school since few children are walking or cycling to school. However, for students entering a classroom immediately following moderate to high level of physical activity from sports or other vigorous activity, MET_EXER_ may be particularly elevated, and thus the post-exercise recovery period should be excluded from the time window used to determine the VR. 

### 3.3. Evaluation of the Methods

The sample of four classrooms in four schools was selected to demonstrate CO_2_-based methods, and results are not necessarily “representative” of elementary school classrooms. Still, results reflect a range of conditions, none of which appear to be exceptional, and they highlight several common issues in using CO_2_-based methods for determining VRs, based on the larger study [36]. First, occupancy levels vary considerably throughout the school day, and “clean” (and ideally prolonged) step-wise increases in occupancy were not seen in any of the classrooms. Instead, teachers arrived before students, students and teachers left for lunch, the number of students varied between morning and afternoon, and students generally entered and left in phases for classes and breaks. Second, VRs in the tested classrooms, all in modern mechanically-ventilated U.S. schools, are below recommended levels of 6.7 L·s^−1^·person^−1^ (assuming default occupancy and children ages 9 and up) [12]. This has been shown in other U.S. schools, e.g., V_0_ averaged 3.6 L·s^−1^·person^−1^ in elementary schools in the southwest U.S. (determined using CO_2_ and the steady-state method [8], and similar results have been reported in other countries [10,41]. Third, VRs were low (around 0.1 h^−1^) in the early morning (and generally the evening) when the building was not occupied, reflecting the shutdown of the ventilation system, and possibly a “tight” building envelope (Tightness was not measured in this study). Such energy and cost-savings measures, taken in the design and operation of many modern buildings, were anticipated for the EnergyStar buildings (Figure 1C,D), but also were apparent for the two conventional buildings tested (not designed to meet EnergyStar or other energy-certification system; Figure 1A,B). As discussed below, these issues have important consequences for VR estimates, which are below by method.

Often, the steady-state CO_2_ concentration C_S_ in classrooms may not be reached, and C_S_ can change over time, a result of low VRs and dynamic occupancy patterns. This was demonstrated in two of the classrooms (Figure 1A,D) that had ‘pointed’ peaks at the maximum CO_2_ concentration. In the two others (Figure 1B,C), CO_2_ trends began to plateau, an indication of the approach of steady-state conditions, but trends never became horizontal. The potential to overestimate VRs when the steady-state concentration is not attained has been recognized for many years, though this method continues to be widely used [26]. For CO_2_ measurements, we do not recommend averaging periods shorter than 5 to 10 min (or the use of instantaneous measurements) for C_S_, especially in schools and where instrumentation is unattended, given instrument noise and the possibility of unrepresentative spikes (e.g., children breathing on sensors). This also applies to CO_2_ measurements used in the build-up and other methods.

For classrooms that have just been vacated, CO_2_ levels tend to decline smoothly and exponentially, and decay and transient mass balance methods give comparable results. For the 2-point decay method (Equation (5)), selecting C_0_ and C_1_ as the maximum and minimum concentrations, respectively, within ±1 h of the nominal times for a 6 h time window, also gave similar estimates of A_D_ as the nominal times. The decay method provided consistent results for evening and early morning time windows using 15-min average CO_2_ measurements and time windows as short as 2 h. However, VRs determined for the unoccupied period did not reflect conditions during the occupied period (see Section 2.2). Thus, decay-rate VRs may be less relevant to health and comfort investigations, although VRs estimated for the unoccupied period may relate to the build-up of emissions associated with building materials and furnishings (e.g., formaldehyde) and they may have energy-related and other implications. 

The build-up method does not require steady-state conditions and can provide consistent results given a step-wise increase in CO_2_ generation rates. The method requires the identification of an appropriate time window, which must exclude changes in occupancy, and specifically the lunch period when students typically leave the classroom. Given patterns observed in the elementary schools, we recommend a nominal time window from 08:00 to 12:00 with flexible start and end times (±1 h) so as to minimize C_0_ and maximize C_1_. The classroom must be continuously occupied during the selected period. This protocol excludes the lunch hour and improves reliability. A 2 h time window was usually sufficient, which may allow analysis of both morning and afternoon build-up periods (again excluding the lunch period). Still, results can remain sensitive to the time window selected. This applied particularly to the ASTM and 3-point methods, which did not obtain consistent results, an unsurprising result given the irregularity of the observed build-up curves. In addition, the 3-point method failed when Equation (9) yielded a negative value of C_S_ (as discussed in Section 2.3). Of the build-up methods, the implicit solution approach (Equations (10)–(12)) is recommended. This approach requires only two CO_2_ measurements, converged rapidly in each classroom tested using the modified Newton-Raphson method and the suggested starting solution, provided good stability, and obtained VR estimates that were more consistent with those of the transient mass balance method. However, this approach requires an estimate of occupancy over the sampling period. Only the morning period was used with the build-up method; longer periods gave poor outcomes due to occupancy changes. Even with the flexible start and stop times suggested, build-up air change rates provided by the implicit method were typically smaller (by 20% to 35%) than those calculated using the transient mass balance method, again, likely due to the irregular occupancy patterns. For each of the build-up methods, a CO_2_ concentration trend that follows the expected build-up curve is a necessary but not sufficient condition of a step-wise increase in occupancy and constant CO_2_ generation rate, assumptions of this method. To confirm results of the build-up method, the CO_2_ trend must follow the expected pattern, occupancy records must show a stepwise increase, and the physical activity level of students in the classroom must be constant. To minimize possible effects of the post-exercise recovery period, the selected time window might be lagged by at least one hour following periods when students undertake moderate to high intensity exercise, e.g., gym class.

The transient mass balance method, which uses a simple fully-mixed model and a straightforward optimization, yielded results that closely fitted observed CO_2_ levels in most cases. When occupancy patterns are irregular and steady-state levels are not achieved, the transient method generally provided larger and more potentially more accurate VRs than those from the steady-state and build-up methods. Because steady-state conditions or knowledge of C_S_ are not required, and because all CO_2_ data are used (not just the measurements that border the analysis time window), the transient mass balance method may provide the most accurate and robust results among CO_2_-based methods for periods when classrooms are occupied. However, discrepancies can result from errors in reported occupancy and unknown activity levels of the children. Still, the overall agreement observed is excellent, demonstrating the feasibility and performance possible when using teacher−reported occupancy and 15-min block data. In most cases, results were not sensitive to the period selected, e.g., VRs for the 08:00 to 14:30 period were generally within 5% of those for the 08:00 to 16:00 period. Greater sensitivity can result if simulated and observed concentrations diverge (e.g., in the afternoon in Figure 1D), but this was the exception. As with the other methods, the time window should not bridge periods in which HVAC operation is changed.

### 3.4. Limitations

Several limitations apply to the methods and results. First, each room is assumed to be a single well-mixed zone that could be characterized by measurements at a central, but single location. Guidance for VR measurements using CO_2_ specifies that CO_2_ concentrations at representative locations should differ by less than 10% [24]. In the test classrooms, measurements that could evaluate this assumption were not obtained, but our experience in schools and other mechanically-ventilated spaces suggests that a central monitoring site is likely to provide measurements representative of the space. This is less likely when the HVAC system is turned off and mixing is reduced; however, the well-behaved decay curves as well as similar VRs determined for other classrooms in the same building at the same time [36] suggest that the CO_2_ measurements remained representative. The single zone model applied to classrooms has other limitations, e.g., classrooms can be connected together and HVAC zones can include hallways and other spaces in the building. In these cases, multizone models and additional parameters, e.g., multiple tracer gases and/or air flow measurements, may be needed to evaluate air change rates between building spaces as well as outdoors. 

Second, measurements also need to be accurate, and errors will increase as CO_2_ levels decrease and approach outdoor levels. We did not evaluate the validity of the single zone assumption, i.e., interzone air and CO_2_ transport were neglected. Third, the steady-state and decay methods assumed that CO_2_ levels in replacement air C_R_ are constant and known (or estimated), and the former method also requires attainment (or near-attainment) of steady-state CO_2_ levels. CO_2_ levels in replacement air should be based on contemporaneous outdoor measurements near the air intake of the space, thus accounting for possible differences in outdoor levels due to CO_2_ emissions from vehicle exhaust and other sources. Fourth, the decay, build-up and transient mass balance methods require measurements taken over a long enough period to observe a meaningful CO_2_ concentration change. This is rarely an issue in classrooms. Fifth, CO_2_ generation rates depend on the physical activity level of children in the classroom as well as the activity just prior to entering the classroom. The true activity level is unknown, may considerably exceed the default value assumed or the range considered, and may vary over time. This can affect VR estimates determined using the simulation, build-up and steady-state methods. Sixth, our analysis of the post-exercise recovery period was modeled, and supporting observations for children are not available. Further analysis of this issue is suggested. Seventh, each of the methods discussed require that the true VR does not change during the time window considered. As mentioned, VRs can vary for many reasons, especially in buildings that are naturally ventilated, that use variable air volume systems, that turn on or off HVAC systems during the time window, or if the infiltration rate varies during the time window (e.g., due to changes in outdoor temperatures and winds). 

Examples were provided for a small set of classrooms with the intention of demonstrating the variability of CO_2_ patterns in schools. The sample is not necessarily representative. All studied classrooms were mechanically-ventilated and in relatively new buildings. We did not address day-to-day variability in the studied classrooms and among classrooms in the school, or quantify the uncertainty of results. However, we discussed uncertainties resulting from variations in occupant heights, weights and activity levels, occupancy data, method variation and the applicability of its assumptions, CO_2_ measurement errors, and the adequacy of mixing, among other factors.

Finally, it can be difficult to evaluate the performance of CO_2_-based and other methods to determine VRs. An independent and “reference” method, e.g., using a different and unique tracer gases or air flow measurements, was not utilized to confirm the results. Even these methods involve many assumptions and few if any intercomparisons of different methods have been conducted in parallel measurements under real-life conditions [15]. While VRs were estimated using information obtained from HVAC drawings, schedules and manufacturer’s material, this information was incomplete and does not necessarily reflect actual performance. In all cases, even the minimum VRs estimated using this information consistently exceeded the in-use VRs determined using the CO_2_-based methods. 

## 4. Conclusions

Ventilation rates are critical parameters that relate to building energy performance and the health and comfort of occupants. Ventilation rates determined using CO_2_-based methods, which are commonly applied, were reviewed and critiqued, with special attention to classroom applications. The methods were demonstrated in primary school classrooms in modern and mechanically-ventilated buildings. As shown in the literature, ventilation rates in the tested classrooms during the school day fell below minimum recommendations. Air change rates during evening and early morning, when the ventilation system was shut down, were far lower (around 0.1 h^−1^). 

VRs determined using transient mass balance methods are flexible, robust and advantageous given the low VRs and dynamic occupancy patterns often found in classrooms. These methods require information pertaining to changes in occupancy, some basic building and occupancy parameters, and numerical methods to estimate air change rates. Several updates to the build-up method were presented, namely, an implicit method to find A_B_ and C_S_ simultaneously using occupancy information, and an adaptive approach to selecting the analysis time window. The utility of the steady-state and build-up methods may be limited in classrooms for several reasons: the steady-state CO_2_ concentration may not be reached, measured or estimated accurately; a step-wise increase in occupancy may not be observed or confirmed; and post-exercise recovery can alter the CO_2_ generation rate, particularly with vigorous physical activity immediately prior to entering a classroom. During unoccupied periods, the decay and transient mass balance methods can provide equivalent results, however, VRs determined for such periods will not be representative of the occupied portion of the school day if the HVAC system is shut-down or a VAV system is utilized and the heating or cooling load changes significantly when occupants leave. In the schools examined, most HVAC systems were shut down immediately after students left. Thus, VRs determined at the end of the school day will likely be of less interest for many types of building investigations. 

If occupancy measurements can be obtained, then VRs determined using transient mass balance methods will provide the most accurate and robust results. Without occupancy measurements, CO_2_ concentration trends must be carefully examined to determine whether the steady-state or build-up method can be used, and to determine an appropriate time window for analysis. Still, sufficient occupancy data should be collected to confirm that the method’s assumptions and results are correct. Better VR measurements would improve a number of applications, including research aimed at understanding the linkage between ventilation and health. 

## Figures and Tables

**Table 1:** Height and weight data, surface area, and estimated CO_2_ generation rate G_P_ by grade level (for children) and age (for adults).

Grade Level	Activity (MET)	Age Range		Weight		Height		Surface Area		CO_2_ Emissions		
		Start (Year)	End (Year)	Boys (kg)	Girls (kg)	Boys (cm)	Girls (cm)	Boys (m^2^)	Girls (m^2^)	Boys (L/min)	Girls (L/min)	Average (L/min)
PK	1.4	4	5	18.4	18.0	108.9	107.7	0.74	0.73	0.149	0.146	0.147
PK–K	1.4	4	6	19.6	19.1	112.1	111.2	0.78	0.77	0.156	0.153	0.155
K	1.4	5	6	20.7	20.3	115.4	114.7	0.81	0.80	0.163	0.161	0.162
K–1	1.4	5	7	21.9	21.5	118.5	118.0	0.85	0.84	0.170	0.169	0.169
1.0	1.4	6	7	23.1	22.8	121.7	121.4	0.89	0.88	0.178	0.176	0.177
1.5	1.4	6	8	24.4	24.3	124.8	124.4	0.92	0.92	0.185	0.184	0.185
2.0	1.4	7	8	25.7	25.7	127.8	127.5	0.96	0.96	0.192	0.192	0.192
2.5	1.4	7	9	27.2	27.4	130.6	130.2	1.00	1.00	0.200	0.200	0.200
3.0	1.4	8	9	28.6	29.1	133.4	132.9	1.04	1.04	0.208	0.209	0.208
3.5	1.4	8	10	30.3	31.0	136.0	135.5	1.08	1.09	0.216	0.217	0.217
4.0	1.4	9	10	32.0	33.0	138.6	138.1	1.12	1.13	0.224	0.226	0.225
4.5	1.4	9	11	34.0	35.1	141.1	141.2	1.16	1.18	0.233	0.236	0.235
5.0	1.4	10	11	36.0	37.2	143.6	144.2	1.21	1.23	0.242	0.246	0.244
5.5	1.4	10	12	38.3	39.4	146.5	147.6	1.26	1.28	0.252	0.256	0.254
6.0	1.4	11	12	40.6	41.6	149.3	151.0	1.31	1.33	0.262	0.267	0.264
6.5	1.4	11	13	43.1	43.7	152.8	153.8	1.36	1.38	0.273	0.276	0.274
7.0	1.4	12	13	45.6	45.7	156.2	156.7	1.42	1.42	0.284	0.285	0.285
7.5	1.4	12	14	48.3	47.5	159.9	158.4	1.48	1.46	0.296	0.292	0.294
8.0	1.4	13	14	51.0	49.2	163.6	160.1	1.54	1.49	0.308	0.299	0.303
9.0	1.4	14	15	56.2	51.9	169.5	161.7	1.64	1.54	0.329	0.308	0.319
10.0	1.4	15	16	60.8	53.8	173.2	162.5	1.73	1.57	0.346	0.314	0.330
11.0	1.4	16	17	64.4	55.1	175.1	162.9	1.78	1.58	0.357	0.317	0.337
12.0	1.4	17	18	67.1	56.2	176.1	163.1	1.82	1.60	0.365	0.320	0.343
Mixed	1.4	5	11	28.8	29.3	129.2	129.4	1.02	1.02	0.203	0.205	0.204
Adult	1.7	20	70	88.2	75.6	176.8	163.3	2.05	1.82	0.500	0.442	0.471

Weight and height data for ages 4 to 18 years using means of monthly data for U.S. children [48]. Weight data for adults (average over ages 20 to 70) based on NHANES 1999–2006 data, taken from Table 8-3, Exposure Factors Handbook 2011 Edition (Final) [49], PK = prekindergarten; K = kindergarten; decimal grades are mixed (e.g., “1.5” is the average of grades 1 and 2, “mixed” is average of kindergarten through 5th grade).

**Table 2:** Summary of tested classrooms, schools, ventilation systems and prevailing weather conditions. Local (airport) temperature and wind speed (mean and range in parentheses) for school day (08:00–15:00) and evening and morning period (18:00–06:00).

ID	Room, School, HVAC, Weather	HVAC Description and Calculated Air Change Rate
S07C4	Large mixed-grade classroom (299 m^3^) in a midsize conventional building (9012 m^2^, 29 classrooms) constructed in 2005. School day: Temp: 13 (11–15) °C Wind speed: 9 (7–11) m/s Eve + Morn: Temp: 10 (9–11) °C Wind speed: 12 (8–15) m/s	Building uses conventional variable air volume (VAV) system with central air handling units (AHUs) and energy recovery (ER). Five ceiling mounted diffusers in the room collectively discharge 142 to 519 L·s^−1^ (minimum of 236 L·s^−1^ during the heating season) with a minimum of 30% outside air (OA). Given building/HVAC data, estimated air change rates are 0.85 to 1.45 h^−1^ in the heating season.
S14C2	Large prekindergarten/ kindergarten classroom (322 m^3^) in a smaller (7430 m^2^, 22 classrooms) and newer building constructed in 2011. School day: Temp: 5 (4–5) °C Wind speed: 5 (4–6) m/s Eve + Morn: Temp: 8 (5–9) °C Wind speed: 7 (5–12) m/s	Classroom uses vertical unit ventilator (UV) with multiple fan speeds, fully adjustable dampers, and rated capacity of 755 L·s^−1^. UV had dirty filters (reportedly changed twice yearly). Assuming a minimum flow of 40% of capacity and a minimum of 30% OA, the air change rate is 1.01 h^−1^.
S19C3	2nd grade classroom (213 m^3^) in a midsize (10,400 m^2^, 36 classrooms) EnergyStar building constructed in 2005. School day: Temp: −11 (−17–−6) °C Wind speed: 6 (3–8) m/s Eve + Morn: Temp: −12 (−17–−8) °C Wind speed: 5 (3–8) m/s	Building uses geothermal heat pumps for each classroom and centralized make-up air (100% OA) discharged through four ceiling diffusers. Design drawings show the maximum OA flow rate to the room of 566 L·s^−1^. Assuming a minimum of 30% of rated flow, the air change rate is 2.87 h^−1^; at maximum flow, the rate is 9.56 h^−1^.
S30C4	Large 4th grade classroom (281 m^3^) in a smaller (5760 m^2^, 21 classrooms) EnergyStar building that has had several expansions since original construction in 1975. Complete building renovation in 2007. School day: Temp: −6 (−7–−4) °C Wind speed: 4 (3–6) m/s Eve + Morn: Temp: −4 (−6–−2) °C Wind speed: 6 (4–7) m/s	Building uses a UV in each classroom with a maximum flow of 519 L·s^−1^ discharged through four ceiling diffusers. Assuming a minimum of 50% of the rated flow and 30% OA, the air change rate is 1.00 h^−1^; at maximum flow, the air change rate is 2.00 h^−1^.

**Table 3:** Estimated air change rates (h^−1^) in four classrooms based on transient mass balance, steady-state, build-up and decay methods.

Period of Day	Method	Conventional Buildings		EnergyStar Buildings	
		S07C4	S14C2	S19C3	S30C4
School day (occupied)					
	Transient mass balance	0.51	0.77	2.42	0.70
	Steady-state	0.52	0.67	1.41	0.80
	Build-up-3 point	0.31	0.77	0.70	0.22
	Build-up-implicit	0.43	0.57	2.10	0.62
	Build-up-ASTM	0.52	0.68	13.19	1.34
Evening (unoccupied)					
	Transient mass balance	0.14	0.18	0.27	0.72
	Decay	0.13	0.13	0.17	0.57
Early morning (unoccupied)					
	Transient mass balance	0.098	0.057	0.113	0.011
	Decay	0.085	0.025	0.322	0.053

## Supplementary Materials

The following are available online at www.mdpi.com/1660-4601/14/2/145/s1, Figure S1: Trends of a child’s activity level and the relative bias, showing the effect of the recovery period following exercise. The instantaneous MET trend is predicted using Equation (19). The average MET trend averages the instantaneous MET levels back to time t = 0. The relative error represents the difference between the average MET level (accounting for the recovery period) and the sedentary MET level (1.4 MET). Three cases are shown. Case 1 uses nominal values of recovery time and exercise activity. Case 2 uses parameters selected to have a “maximal effect” on MET predictions. Case 3 uses parameters selected to have a “minimal effect” on MET predictions. The Supplementary Materials also presents the full solution for the implicit build-up method. The author will gladly share an Excel workbook that clearly demonstrates each of the methods discussed, and a second macro-enabled Excel workbook that demonstrates the calculations and produces the graphics in this paper. Please contact the author.
