# Supplementary material for: Review and Extension of CO2-Based Methods to Determine Ventilation Rates with Application to School Classrooms
Source: Int J Environ Res Public Health. 2017 Feb 4;14(2):145. doi: 10.3390/ijerph14020145 (PMC5334699; doi:10.3390/ijerph14020145)
Supplement: Supplementary file 1 [file ijerph-14-00145-s001.pdf]

Supplementary Materials: Review and Extension of CO_2-Based Methods to Determine Ventilation Rates with Application to School Classrooms

Stuart Batterman

1. Trends of Activity Levels Following Exercise: Sensitivity Analysis

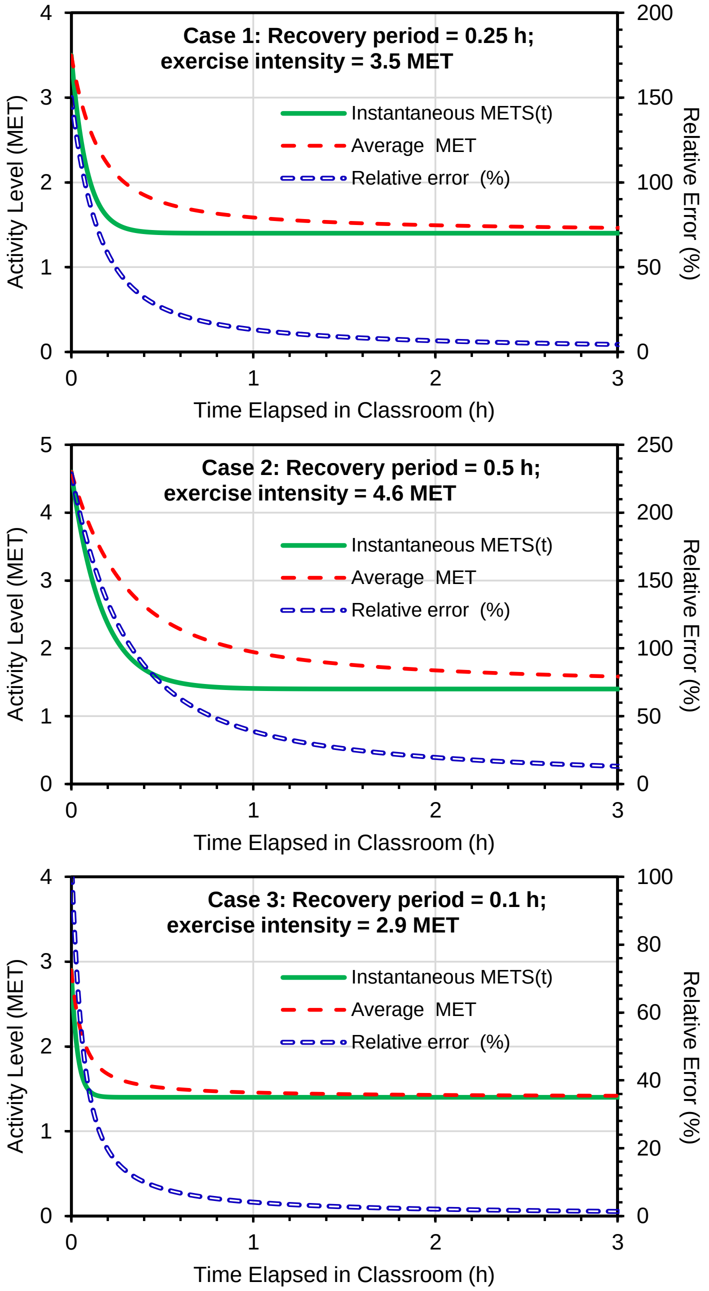

Figure S1. Trends of a child's activity level and the relative bias, showing the effect of the recovery period following exercise. The instantaneous MET trend is predicted using Equation (19). The average MET trend averages the instantaneous MET levels back to time t = 0. The relative error represents the difference between the average MET level (accounting for the recovery period) and the sedentary MET level (1.4 MET). Three cases are shown. Case 1 uses nominal values of recovery time and exercise activity. Case 2 uses parameters selected to have a "maximal effect" on MET predictions. Case 3 uses parameters selected to have a "minimal effect" on MET predictions.

2. Solution for the Implicit Build-Up Method

If the steady-state concentration is known, then the buildup air change rate A_B (h^{-1}) can be estimated using two sequential CO_2 measurements:

$$A_B = 1/\Delta t \ln\{(C_S - C_0)/(C_S - C_1)\} \quad \text{(S1)}$$

where Δt = period between C_0 and C_1 measurements (h), C_S = steady-state concentration (ppm), and C_0 and C_1 = CO_2 concentrations measured at start and end of the observation period, respectively (ppm). However, C_S is not usually known. An implicit approach can be used simultaneously solve for the air change rate and the steady-state concentration. Given an initial estimate of the air change rate, $\hat{A}_B$, an estimate of the steady-state concentration is:

$$\hat{C}_S = 6 \times 10^4 \text{ n } G_P/(V \hat{A}_B) + C_R \quad \text{(S2)}$$

Equations (S1) and (S2) can be solved simultaneously. To simplify, let $K = 6 \times 10^4$ n G_P/V. Then Equation (2) becomes:

$$\hat{C}_S = K/\hat{A}_B + C_R \quad \text{(S3)}$$

Substituting Equation (S3) into Equation (S1):

$$\hat{A}_B = 1/\Delta t \ln\{(K/\hat{A}_B + C_R - C_0)/(K/\hat{A}_B + C_R - C_1)\}$$

Solving for the root:

$$\hat{A}_B \Delta t = \ln\{(K/\hat{A}_B + C_R - C_0)/(K/\hat{A}_B + C_R - C_1)\}$$

$$0 = \exp(\hat{A}_B \Delta t) - (K/\hat{A}_B + C_R - C_0)/(K/\hat{A}_B + C_R - C_1) \quad \text{(S4)}$$

Equation (S4) does not have an analytical solution. We obtained solutions using a modified Newton-Raphson method, which expresses successive estimates of the unknown x by evaluating the function $f(X_N)$ and its first derivative $f'(X_N)$. A dampening factor d is incorporated to moderate swings that can cause divergence:

$$x_{N+1} = X_N - df(x_N)/f'(x_N)$$

The function $f(x_N)$ is given as Equation (S4); the first derivative is:

$$\frac{\partial}{\partial x}\left(\exp(x\,T) - \frac{\frac{K}{x} + R - A}{\frac{K}{x} + R - B}\right) = -\frac{K\left(-A + \frac{K}{x} + R\right)}{x^2\left(-B + \frac{K}{x} + R\right)^2} + \frac{K}{x^2\left(-B + \frac{K}{x} + R\right)} + T\,e^{T\,x} \quad \text{(S5)}$$

where $T = \Delta t$; $R = C_R$; $A = C_0$; $B = C_1$; and $x = \hat{A}_B$. Since C_S must exceed C_1, $\hat{A}_B$ is bounded:

$$\hat{A}_{B,MAX} < 6 \times 10^4 \text{ n } G_P/\{V\, C_1\, (1 - C_R/C_1)\} \quad \text{(S6)}$$

To solve this system, the modified Newton-Raphson method was used with a starting estimate of $0.999 \times \hat{A}_{B,MAX}$, and a dampening factor d = 0.25. Typically, convergence was attained rapidly in nearly all cases, e.g., fewer than 20 iterations of Equation (S5) were required.

© 2017 by the authors; licensee MDPI, Basel, Switzerland. This article is an open access article distributed under the terms and conditions of the Creative Commons by Attribution (CC-BY) license (http://creativecommons.org/licenses/by/4.0/).
